# The Shaker Potassium Channel Is No Target for Xenon Anesthesia in Short-Sleeping *Drosophila melanogaster* Mutants

**DOI:** 10.1100/2012/373709

**Published:** 2012-06-18

**Authors:** C. Schaper, J. Höcker, R. Böhm, T. Roeder, B. Bein

**Affiliations:** ^1^Department of Anaesthesiology and Operrative Intensive Care Medicine, University Hospital Schleswig-Holstein, Campus Kiel, Schwanenweg 21, 24105 Kiel, Germany; ^2^Institute of Clinical and Experimental Pharmacology, University Hospital Schleswig-Holstein, Campus Kiel, Arnold-Heller-Straße 3, Haus 30, 24105 Kiel, Germany; ^3^Department of Zoophysiology, CAU Kiel, Olshausenstraße 40, 24098 Kiel, Germany

## Abstract

*Background*. Xenon seems to be an ideal anesthetic drug. To explore if next to the antagonism at the NMDA-receptor other molecular targets are involved, we tested the xenon requirement in short sleeping *Drosophila* shaker mutants and in *na*[*har* ^38^]. *Methods*. The *Drosophila melanogaster* strains wildtype Canton-S, *na*[*har* ^38^], *sh* ^102^ and *sh* ^*mns*^, were raised and sleep was measured. Based on the response of the flies at different xenon concentrations, logEC50 values were calculated. *Results*. The logEC50-values for WT Canton-S were 1.671 (1.601–1.742 95%-confidence intervall; *n* = 238; *P* versus *sh* ^102^ > 0,05), for *sh* ^*mns*^ 1.711 (1.650–1.773; *n* = 242; *P* versus WT Canton-S > 0,05). The logEC50-value for *sh* ^102^ was 1.594 (1.493–1.694; *n* = 261; *P* versus *sh* ^*mns*^ > 0.05). The logEC-value of *na*[*har* ^38^] was 2.076 (1.619–2.532; *n* = 207; *P* versus *sh* ^*mns*^ < 0.05, versus *sh* ^102^ < 0.05, versus WT Canton-S < 0.05). *P* values for all shaker mutants were *P* > 0.05, while *na*[*har* ^38^] was found to be hyposensitive compared to wildtype (*P* < 0.05). *Conclusions*. The xenon requirement in *Drosophila melanogaster* is not influenced by a single gene mutation at the shaker locus, whereas a reduced expression of a nonselective cation channel leads to an increased xenon requirement. This supports the thesis that xenon mediates its effects not only via an antagonism at the NMDA-receptor.

## 1. Introduction

In the last decade, the noble gas xenon has been increasingly used for anesthesia because of its low blood-gas partition coefficient, its organ protective effects, and its hemodynamic stability [1]. Xenon mediates its effects via antagonism of the *N-Methyl-D-Aspartate* (NMDA) receptor, which is one of the glutamate-activated ion channels. The NMDA receptor is linked with synaptic functions like memory, pain and learning [2]. Next to this well-known mechanism at the NMDA receptor, recent studies have focused on other molecular targets for xenon anesthesia. The inhibition of non-NMDA receptors [3], cellular pathways like calcium-homeostasis [4], or the activation of potassium channels is described [5]; so there seems to be a complex mixture of targets for xenon anesthesia including widely spread ion channels.

The fruit fly *Drosophila melanogaster* possesses a complex nervous system organized into circuits and also homologue ion channels can be found. Like humans, fruit flies exposed to volatile anesthetics run through several states of anesthesia ending in an immobile state, also the EC 50 values are comparable [6]. Many of the ion channels in *Drosophila melanogaster* are conserved across organisms. The *Drosophila* shaker-related gene has its vertebrate counterpart in Kv1 [7]. The neuronal channel NALCN has a homology in *Drosophila* named alpha1U [8]. The *Drosophila melanogaster* shaker mutants exhibit a gene mutation in the shaker locus, which encodes for a voltage-gated potassium channel. This mutation becomes manifest in a short-sleeping phenotype, so these strains sleep significantly less than the wildtype [9]. 

In a previous study, we have shown that these short sleeping *Drosophila melanogaster* shaker mutants had an increased anesthetic requirement of the volatile anesthetics isoflurane and sevoflurane [10]. The mutant strain *na*[*har* ^38^] (narrow abdomen; halothane anesthesia resistance), which exhibits a reduced expression of the nonselective cation neuronal channel NALCN [11], is known to be resistant to halothane, methoxyflurane, chloroform, and trichloroethylene.

To more specifically explore the role of ion channels in xenon anesthesia and to identify new molecular targets for xenon, we hypothesized that mutations in the shaker potassium channel or the NALCN neuronal channel lead to a modified xenon requirement in *Drosophila melanogaster.*

## 2. Methods 

### 2.1. Animals

This study was approved by the local animal investigational committee (Christian Albrechts University Kiel). *Drosophila melanogaster* was bred in the laboratory at 21°C, 68% humidity, on yeast, dark corn syrup, and agar food. For determination of sleep and wakefulness, male and female fruit flies were used in equal numbers. To exclude age-associated effects, only young flies (≤2 weeks) were tested for all experiments [12]. *Drosophila* stocks used were *sh* ^*mns*^, *sh* ^102^, *na*[*har* ^38^], and wild-type Canton-S. To remove modifiers, stocks were consequently outcrossed for at least five rounds to Canton-S background as described before [13]. 

### 2.2. Determination of Locomotor Activity

Sleep and wakefulness were determined from individual fruit flies placed in a *Drosophila* activity monitor system (DAMS, Trikinetics, Waltham, MA, USA) at constant environmental conditions. Male and female flies were used in equal numbers. Activity measurement was recorded for consecutive one-minute periods for one week after one day of adaptation and analyzed with custom-designed software developed in our laboratory. As described before, sleep was defined as any period of uninterrupted behavioural immobility (0 counts per minute) lasting >5 minutes [14]. The total duration of sleep episodes was then calculated exactly to the minute.

### 2.3. Measurement of Anesthetic Sensitivity

Anesthetic sensitivity was tested in a custom-made *Drosophila* anesthesia chamber (*V* = 200 mLs) connected to xenon with a constant flow of 1 L·min^−1^. For each experiment, at least 10 young (≤2 weeks) wild-type or mutant strain fruit flies were placed inside the chamber and exposed to xenon concentrations from 20 to 80%. After a 10-minute exposure, the chamber was rotated and shaken for 2 seconds under the control of a motor, which caused the flies to fall from their current position to the bottom of the chamber. Anesthetized flies stayed immobile. With this accepted method to deprive sleep [14], we were able to distinguish between physiological sleep and anesthesia. The numbers of mobile and immobile flies were counted by a blinded observer, whereas a convulsion was not considered a movement. The results were recorded for subsequent statistical analysis. All experiments were carried out at constant environmental temperature of 21^° ^C, and concentrations of xenon were continuously monitored at the chamber outflow with an oxygen monitor (Dräger Medical AG & Co. KG, Lübeck, Germany). 

### 2.4. Statistical Analysis

Student's *t*-test was used to assess statistically significant differences for periods of sleep and wakefulness between *Drosophila* strains. Based on the response of the flies at different concentrations of xenon, concentration-response curves were generated. Data were imported into GraphPad Prism (GraphPad Software, San Diego, CA, USA), and a nonlinear regression analysis was performed for log(dose) versus normalized response with variable Hill slope. Resulting logEC50 was further analyzed with one-way ANOVA and Bonferroni posttest. The half-maximum effective concentration (EC_50_) values and 95% confidence intervals were calculated and compared for statistically significant differences. 

## 3. Results 

The daily sleep amount in *Drosophila melanogaster* WT *Canton-S* (*n* = 64) was 965 ± 15 minutes (mean ± SEM), *sh* ^*mns*^ (*n* = 32) slept 595 ± 45 minutes, *sh* ^102^ (*n* = 32) slept 764 ± 39 minutes. Compared to WT *Canton-S,* both Shaker mutants were mini-sleepers (*P* < 0.01), so the short-sleeping phenotype was expressed as expected. The mutant strain *na*[*har* ^38^] (*n* = 32) slept 998 ± 29 minutes, so the daily sleep amount was comparable to the wildtype, as it was described before.

The anesthetic requirement of xenon was calculated from the response of the flies at different concentrations of xenon (20–80%). 

**Figure 1:**
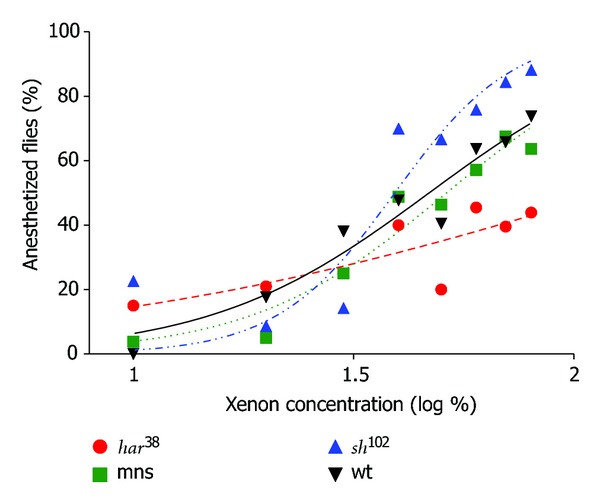
Percentage of anesthetized flies at different concentrations of xenon. Dots show the actual measured data points; lines show the best-fit sigmoidal curve for these data points. Note that the *y*-axis has a logarithmic scale.

**Figure 2:**
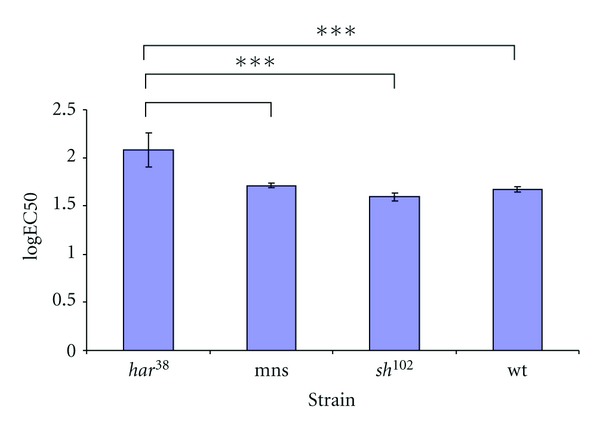
logEC50 of xenon for different strains of flies (mean ± SE). The logEC50 in *na*[*har* ^38^] flies is significantly higher than in the other three groups.

The logEC50-values for WT Canton-S were 1.671 (1.601–1.742 95%-confidence intervall; *n* = 238; *P* versus *sh* ^102^> 0.05), for *sh* ^*mns*^ 1.711 (1.650–1.773; *n* = 242; *P* versus WT Canton-S > 0,05). The logEC50 value for *sh* ^102^ was 1.594 (1.493–1.694; *n* = 261; *P* versus *sh* ^*mns*^ > 0.05). So there was no statistically significant difference in the xenon requirement of both shaker stocks compared to the wildtype. The logEC value of *na*[*har* ^38^] was 2.076 (1.619–2.532; *n* = 207; *P* versus *sh* ^*mns*^ < 0.05, versus *sh* ^102^ < 0.05, versus WT Canton-S < 0.05). So *na*[*har* ^38^] was found to be hyposensitive to xenon compared to all other tested strains. Fitted curves are shown in Figure 1, and logEC50 values are shown in Figure 2. Xenon had a significant higher EC50 value in *na*[*har* ^38^] flies. 

## 4. Discussion 

Whereas intravenous anesthetics act via specific receptor-ligand interactions, other drugs like the volatile anesthetics or the inert gas xenon act via less well-known pathways. In contrast to the volatile anesthetics, the effects of xenon were mainly attributed to the NMDA receptor subtype of the glutamate receptors [15]. Xenon is known to be an antagonist at the NMDA-receptor, while it has only little effects on the GABA-A receptor [2]. Younger molecular studies focused on other molecular targets for xenon anesthesia: xenon preconditioning was discovered to be dependent on the activation of adenosine triphosphate-sensitive potassium channels [5]. In *Caenorhabditis elegans,* a non-NMDA-receptor was required for effects of xenon anesthesia [3]. In 2004, Gruss et al. described the two-pore domain potassium channel TREK-1 as a novel target for xenon anesthesia [16]. These channels are members of the two-pore-domain potassium channel family. For this reason, we chose well-known *Drosophila* strains with a point-mutation in a gene, which encodes for the alpha subunit of a tetrameric voltage-dependent potassium channel and which is responsible for membrane repolarization after an incoming action potential [17]. We used the two strains *sh* ^*mns*^ and *sh* ^102^, which are strong alleles of the shaker gene with loss-of-function proteins [6]. Electrophysiological and molecular studies found out that in these animals the shaker potassium channel is completely not expressed [18, 19], so we were able to avoid an interaction of xenon with reduced Shaker currents, as they appear in weak alleles. 

For the volatile anesthetics isoflurane, and sevoflurane, we described an increased requirement for these drugs in *Drosophila* shaker mutants, due to this single nucleotide mutation [10]. In the present study, we found no statistically significant difference in the anesthetic requirement of xenon in *sh* ^*mns*^ and *sh* ^102^. Therefore, it might be concluded that the shaker potassium channel is no target for xenon anesthesia in *Drosophila melanogaster* or at least that the action of xenon on the shaker channel has no consequences for anesthetic potency. As a matter of course, the effects of xenon could be caused by the activation of other potassium channels like *shal* or *shab*.

In the next step, we focussed on the *na*[*har* ^38^] strain. These flies have reduced expression of the nonselective neuronal ion channel NALCN, which is permeably for sodium-, potassium-, and calcium ions, and which contributes to resting sodium permeability [11]. In the absence of anesthetics, these flies show a characteristic walking behavior, although they are viable and fertile [20]. In *na*[*har* ^38^] fruit flies used in this study, a reduced expression of the NALCN ion channel provoked altered sensitivities to halothane, isoflurane, and sevoflurane [10, 21, 22]. For xenon, we demonstrated an increased anesthetic requirement compared to wild-type and shaker strains. Apart from the effects of potassium, this might be a consequence of a changed basal excitability of the nervous system due to changed sodium or calcium permeabilities as a result of modified NALCN channels. The calcium homeostasis is especially part of the theory which tries to explain the anesthetic effects of volatile anesthetics. 

In clinical concentrations, xenon and other anesthetic gases inhibit the calcium ATPase pump activity [23], which is responsible for the maintenance of the calcium concentration gradient. Assumed that xenon interacts with the glutamate-dependent NMDA receptor, it is conceivable that the known process of the calcium influx and the sodium and potassium efflux after a depolarization at the NMDA-receptor is hampered by a changed calcium and/or sodium homeostasis. 

*Drosophila melanogaster* is a frequently utilized model for studies investigating the mechanisms of anesthesia because of its homologous ion channels, receptors, or neurotransmitters [8], although there are limitations of our study. The relative short exposure of 10 minutes of xenon anesthesia may neglect other pharmacodynamic effects, but in view of the low blood-gas partition coefficient of xenon, it is acceptable to assume that the flies reached a steady-state. Another limitation of our data is the choice of the anesthetic endpoint: looking at another endpoint may lead to different results, like it was shown for an anesthesia with halothane in *na*[*har* ^38^] [21, 22]. 

Our study shows that a single gene mutation in a voltage-gated potassium channel has no effect on the xenon requirement in *Drosophila melanogaster*, whereas a mutation in the nonselective ion channel NALCN actually has. This leads to the assumption that the shaker potassium channel is not a molecular target for xenon anesthesia.
